# Visualizing Genotypic and Developmental Differences of Free Amino Acids in Maize Roots With Mass Spectrometry Imaging

**DOI:** 10.3389/fpls.2020.00639

**Published:** 2020-05-25

**Authors:** Kelly C. O’Neill, Young Jin Lee

**Affiliations:** Department of Chemistry, Iowa State University, Ames, IA, United States

**Keywords:** mass spectrometry imaging, maize root, amino acids, nitrogen assimilation, nitrogen transportation, on-tissue derivatization

## Abstract

Amino acids are essential biological compounds in plants as they store nitrogen, an essential nutrient, and are the building blocks for proteins that drive biological activity. Amino acids have been studied using a wide variety of analytical techniques in different plant systems, however, mass spectrometry imaging (MSI) is a particularly useful technique as it allows for the simultaneous collection of both chemical and spatial information. In this work, matrix-assisted laser desorption/ionization (MALDI)-MSI is used to study the different localization of free amino acids in the roots of maize inbred lines B73 and Mo17 and their reciprocal hybrids. Because amino acids are difficult to detect in mass spectrometry, especially directly on tissues, a chemical derivatization protocol is utilized to increase the ionization efficiency and improve their detection. We report differences in both abundance and localization of amino acids in B73 and Mo17 maize roots and suggest the hybrids show evidence of inheriting characteristics from both parents. Most genotypic differences are found in the cross-sections near the seed (∼2 cm away) at a later stage of development (10–11 cm in length). Here, B73 has lower amino acid abundance localized primarily to the center of the roots for most amino acids, while Mo17 has much higher abundance localized mainly to the root cortex. This difference in localization is minimized when grown in ammonium ion rich conditions. Roots grown in the presence of ^15^N-ammonium ions provided additional insight about the amino acid synthesis. The localization of some amino acids, particularly leucine/isoleucine and glutamine, is not affected by the addition of nitrogen and is consistent regardless of the nitrogen source, either from the seeds (^14^N-labeled) or environment (^15^N-labeled). Nitrogen uptake from the environment is confined to glutamine, asparagine, and alanine, consistent with their roles in amino acid storage and transportation.

## Introduction

Nitrogen is an essential nutrient for crop plants that has a major impact on crop production and yields. Nitrogen has an important role in plant growth and development; therefore, understanding nitrogen uptake and metabolism can help determine how to best and most efficiently care for plants (Lemaître et al., 2008). Amino acids are responsible for the storage and transportation of nitrogen in plants and are an important aspect of nitrogen metabolism (Pratelli and Pilot, 2014). Recently, maize roots have been used as a model species for biological studies (Paschold et al., 2010); studying roots at their early stage can provide numerous benefits. The emergence of the primary root allows early morphological, histological, and physiological analysis of the seedling, while the fast germination and growth of maize seedlings in laboratory environments allow for high-throughput experiments under controlled and standardized conditions (Paschold et al., 2010). Studying the spatial arrangement of amino acids in maize roots can shed light on nitrogen assimilation and transportation in early plant development.

Mass spectrometry imaging (MSI) has become a valuable analytical tool to visualize metabolites such as lipids and small molecules directly on plant tissues (Lee et al., 2012; Sturtevant et al., 2016). Matrix-assisted laser desorption ionization (MALDI)-MSI is a commonly used technique, particularly for cellular and sub-cellular resolution imaging, due to its combination of high-spatial resolution, high sensitivity, and chemical versatility. Recently, MALDI platforms have been optimized to reach pixel sizes from 1 to 10 μm (Zavalin et al., 2013; Korte et al., 2015), which enables the study of biological tissue at the cellular and even sub-cellular level. Recent advances in t-MALDI-2 have even allowed for spatial resolutions of less than 1 μm (Niehaus et al., 2019). Visualizing detailed metabolite information at this scale can offer unprecedented details in terms of metabolite composition and localization which can be crucial for elucidating their biological roles.

Our group has developed a MALDI-MSI platform that allows for 5–10 μm high-spatial resolution. This setup has been used for various applications including the visualization of numerous different metabolites in maize leaves (Korte et al., 2015; Dueñas et al., 2017), seeds (Dueñas et al., 2016; Feenstra et al., 2017a), and roots (Feenstra et al., 2017b). The work of Dueñas et al. has applied this platform to show that the fatty acyl localizations of some thylakoid membrane lipids such as PG 32:0 are different depending on the genotype of maize. Additionally, the hybrid maize exhibit the characteristics resembling that of the maternal parent (maternal inheritance) (Dueñas et al., 2019). Previous work out of our group has also shown the benefits of utilizing various chemical derivatization reactions to enhance the ion signals for metabolites with certain functional groups. This strategy of using multiple different chemical derivatizations on adjacent tissue sections has allowed for expanded metabolite coverage (Dueñas et al., 2019).

In this work, we used MALDI-MSI to visualize amino acids in roots of two agronomically important inbred lines of maize, B73 and Mo17, and their reciprocal hybrids, B73 × Mo17 (BxM) and Mo17 × B73 (MxB). We focus primarily on the differences in amino acid localization and abundance between the genotypes and at different developmental stages of the root. Amino acids play an essential role in plant biology; they are the building blocks for proteins and have influence in many biochemical pathways relating to growth, development, stress resistance, and signaling (Hildebrandt et al., 2015). Many research efforts have been made previously on the role of amino acids in maize, especially how they relate to nitrogen assimilation and transportation. Employed methods in these studies include colorimetry, high performance liquid chromatography (HPLC), mass spectrometry, and various assays (Fentem et al., 1983; Toro et al., 2003; Seebauer et al., 2004). Despite extensive research in this area, very little is known about the localization of amino acids in maize. Our group has done some work showing the distribution of amino acids in the maize seed during germination, however, this was rather limited due to low ion signals (Feenstra et al., 2017a).

The current study aims to determine the abundance and distribution of amino acids in maize roots under various conditions and find evidence for how hybrid maize inherit molecular characteristics relating to amino acid localization from their parents. In addition to the aforementioned benefits of working with maize roots as a model system, there are additional advantages for this particular study focusing on the early stage of root development. Once vegetative tissue begins to develop and photosynthesis starts, the transportation of nitrogen and amino acids becomes more complex. Overall, focusing on the early development of the root simplifies the study. It has been established that Mo17 has more abundant amino acids in its seeds than B73 (Feenstra et al., 2017a), but it was not well known how that is translated to the hybrids, especially as the seedlings germinate and develop. As amino acids are not easily detectable with MALDI-MSI, derivatization is often necessary in order to increase their ionization efficiency. Coniferyl aldehyde (CA) has previously been used to derivatize primary amines, including amino acids, in MALDI-MSI experiments (Seebauer et al., 2004; Manier et al., 2014; Esteve et al., 2016). This derivatization strategy is utilized here to investigate amino acid differences in B73, Mo17, and their reciprocal hybrid maize genotypes.

## Materials and Methods

### Materials

Gelatin from porcine skin (300 bloom) was purchased from Electron Microscopy Sciences (Hatfield, PA, United States). 4-hydroxy-3-cinnamaldehyde (coniferyl aldehyde; CA), deuterated alanine, ammonium chloride, and 98 atom percent ^15^N ammonium chloride were purchased from Sigma Aldrich (St. Louis, MO, United States). Potassium acetate was purchased from Fisher Scientific (Hampton, NH). The gold sputter target was purchased from Ted Pella, Inc. (Redding, CA, United States). B73, Mo17, MxB, and BxM maize seeds were obtained courtesy of Dr. Marna Yandeau-Nelson at Iowa State University.

### MALDI Sample Preparation

B73, Mo17, BxM, and MxB maize seeds were grown using a method described previously (Feenstra et al., 2017b). A row of maize seeds were placed along the edge of two wetted paper towels stacked on top of one another. The paper towels were wetted with either water, 10 mM ammonium chloride, or 10 mM ^15^N labeled ammonium chloride depending on the experiment. The seeds were then rolled up in the paper towels tightly enough to keep them in place. The paper towel rolls were then placed in a beaker filled with water, 10 mM ammonium chloride, or 10 mM ^15^N labeled ammonium chloride for the isotope labeling experiments. The beaker was placed in the dark while the seeds began to grow and was monitored to make sure the paper towel stayed moist throughout growth. The roots were harvested when the length of the primary root was 2.5–3 cm, 6–7 cm, or 10–11 cm as measured from the tip of the root. The length of the root at harvesting differed depending on the experiment and is stated in each section of the results.

Once the roots reached the desired length, a razor blade was used to cut the root about 2 cm below the seed. This area of interest was embedded in a 10% w/v gelatin solution and flash frozen in liquid nitrogen. The area of interest was about 2 cm from the seed regardless of the stage of development, except in the case where multiple positions on the same root were embedded. The embedded roots were stored at −20°C and allowed to thermally equilibrate. The root tissue was cryo-sectioned (CM 1850, Leica Microsystems; Buffalo Grove, IL, United States) to 10 μm thickness, collected with Cryo-Jane tape (Leica Biosystems), and attached to a pre-chilled glass slide. The prepared slides were stored at −80°C until use, when they were placed on an aluminum block stored at the same temperature and vacuum dried. The dried sample tissues were derivatized using a TM sprayer (HTX Technologies, LLC, Chapel Hill, NC, United States). A 20 mg/mL solution of CA was used for derivatization at a flow rate of 0.03 mL/min and passing 8 times over the sample tissue. After derivatization, the tissue sections were sprayed with 6.5 mM potassium acetate using the same TM sprayer method. This was done to ensure the formation of potassium adducts while limiting protonated or sodiated adducts. They were then subject to matrix deposition by sputter coating (108 Auto Sputter Coater, Ted Pella Inc., Redding, CA, United States) gold at 40 mA for 20 s. One set of experiments incorporated deuterated alanine as an internal standard. The internal standard (5 mM) was sprayed onto the sample using the same TM sprayer method as previously described.

### Mass Spectrometry Imaging Analysis

A MALDI linear ion trap-Orbitrap instrument (MALDI-LTQ-Orbitrap Discovery; Thermo Finnigan, San Jose, CA, United States) was used to collect all MSI data. The instrument was modified to incorporate an external 355 nm frequency tripled Nd: YAG laser (UVFQ; Elforlight, Daventry, United Kingdom). Tuneplus and XCalibur (Thermo Finnigan) were used to develop the mass spectrometry method and acquire data, respectively. Mass spectra were acquired in positive ion mode with the Orbitrap mass analyzer for a scan range of *m/z* 100–1000.

MS images were generated using ImageQuest (Thermo Finnigan) and MSI Reader (Robichaud et al., 2013) with a mass window of ±0.003 Da. Serial tissue sections were prepared as previously described and used for MS/MS analysis. MS/MS was done using the ion trap mass analyzer and were analyzed with a mass window of 1.0 Da and normalized collision energy of 35 were used.

### LCMS Sample Preparation and Analysis

Maize roots of each genotype were grown in the same manner as previously described for the MALDI-MSI analysis. Once the roots were 10–11 cm in length, they were flash frozen in liquid nitrogen, homogenized, and stored at −80°C until analysis. Only the top 4 cm of each root was homogenized (2 cm on either side of the portion embedded for MALDI). This was done in an attempt to include the portion of the root that would most resemble the imaged root sections. In order to ensure there was sufficient tissue for good analyte signals, two roots of each genotype were combined for each biological replicate. Amino acids were extracted and simultaneously derivatized with 500 μL of 20 mg/mL CA in methanol. They were vortexed for 10 min and then centrifuged at 14,000 rpm for 10 min. 100 μL of the supernatant was aliquoted out and dried down. They were then reconstituted in 100 μL of 50:50 methanol:water.

Positive mode LCMS analysis was done on an LCMS 2020 single quadrupole mass spectrometer (Shimadzu, Kyoto, Japan). 1 μL of each sample was injected onto a 4.6 × 150 mm Agilent XDB C18 column with 1.8 μm particle size. Solvent A was water with 0.1% formic and solvent B was acetonitrile with 0.1% formic acid. A flow rate of 600 μL/min was used along with the following gradient: 0% B for the first 2 min, up to 30% B over the next 18 min, 100% B over 2 min, and back down to 0% B after another 2 min, where it was held for an additional 9 min. This gradient was first used by Manier et al. to separate CA derivatized amino acids and neurotransmitters (Manier et al., 2014). Chromatograms were extracted for each of the detected amino acids and the corresponding peaks were integrated.

## Results and Discussion

### Coniferyl Aldehyde Derivatization

**SCHEME 1:**
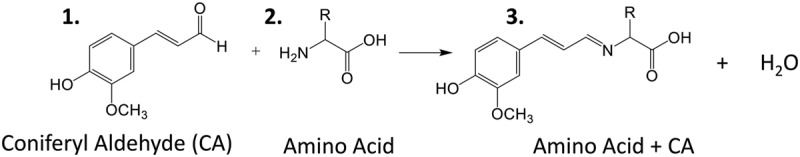
Reaction scheme for the on-tissue derivatization of an amino acid with coniferyl aldehyde.

**FIGURE 1:**
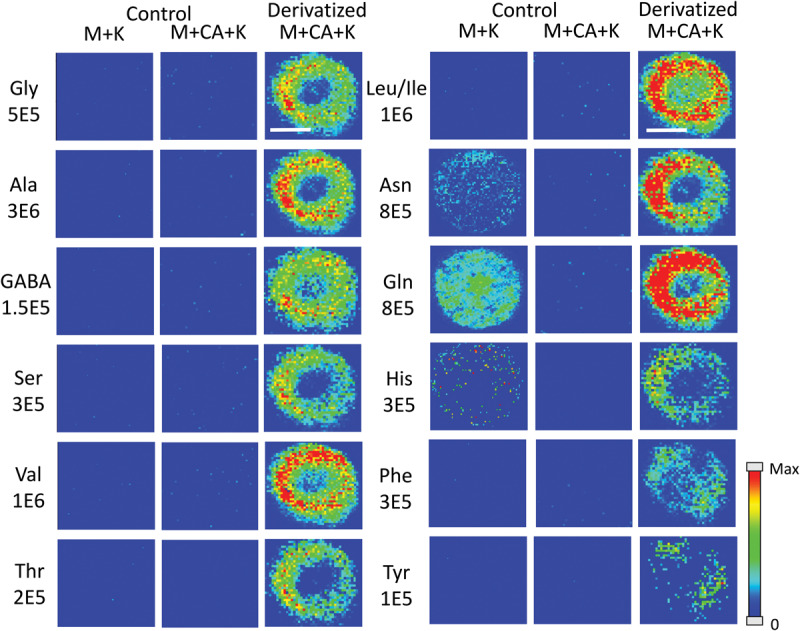
MS images for the 12 amino acids detected in Mo17 maize roots. The first 2 columns are control roots without CA derivatization and the last column is with CA derivatization. “M + K” indicates the non-derivatized amino acids detected as potassium ion adduct. “M + CA + K” indicates the derivatized amino acids detected as potassium ion adduct. The numbers below each amino acid label correspond to the maximum intensity scale used to produce the false color image. The scale bar is 500 μm.

Amino acids are important biological molecules that have numerous roles in plant growth and development. However, due to poor ionization efficiency, amino acids are difficult to study by mass spectrometry. For this reason, coniferyl aldehyde (CA) was employed as a chemical derivatization reagent to modify the amino acids and increase their ionization efficiency (Manier et al., 2014; Esteve et al., 2016). The reaction scheme is shown in Scheme 1. CA (1) reacts with primary amines, such as amino acids (2), and forms a product with an imine moiety that improves ionization efficiency (3). Figure 1 shows representative MALDI-MS images for 12 amino acids visualized in the maize root. A total of 16 amino acids were detected with the derivatization (14 proteogenic and 2 non-proteogenic) but only those with high enough signal are displayed. As leucine and isoleucine are isomers, they are not distinguishable by mass and are shown as a mixture of the two. Without derivatization with CA, only 3 amino acids are detected in positive mode (asparagine, glutamine, and histidine) and there is no signal present at the derivatized masses. Once CA derivatization is utilized, there is significant signal improvement to generate ion images for 12 amino acids. Four more amino acids (tyrosine, glutamic acid, proline, and aminobutenoic acid) could be also detected with derivatization but had very low ion signals and could not provide meaningful localization information. This implies the utility of the adopted derivatization technique for the study of amino acids in maize root tissues. The reaction efficiency of glutamine is 80∼90%. This was calculated from dividing the ion signal of derivatized glutamine by the sum of the ion signals for derivatized and underivatized glutamine. All amino acids were identified based on the exact mass of the derivatization product and three of the most abundant, alanine, valine, and leucine, were also confirmed by MS/MS and comparing with that of a CA derivatized standard.

MSI was used to explore amino acids in maize roots of inbreds B73, Mo17, and their reciprocal hybrids. Differences in abundance and localization as the roots develop and between genotypes were studied. Maize roots have a unique architecture which allows for an efficient uptake of water and nutrients (Hochholdinger, 2009). Bright-field microscope images and anatomical assignments are shown in Supplementary Figure S1 for the cross-section of a B73 root. Maize roots exhibit a central vascular cylinder composed of the pith, xylem vessels, and the pericycle (the outermost cell layer of the inner cylinder). The ground tissue is made up of a single endodermis layer, multiple layers of cortex tissue, and a single epidermis cell layer.

### Developmental Changes in the Localization of Amino Acids in Maize Roots

**FIGURE 2:**
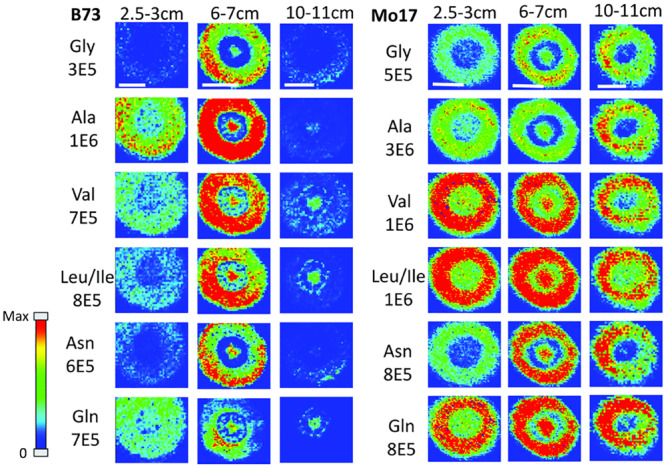
MS images of amino acids in B73 (left) and Mo17 (right) maize roots cryosectioned at 2 cm below the seed for each different stage of development. The numbers below each amino acid label correspond to the maximum intensity scale used to produce false color image. The scale bar is 500 μm.

In order to determine how amino acids change in maize roots throughout development, roots were harvested at three different times. Harvesting times were when the total length of the root measured from the tip was between 2.5–3 cm, 6–7 cm, and 10–11 cm. These roots were each embedded and cryosectioned at about 2 cm away from the seed regardless of the developmental stage. The resulting images are shown in Figure 2 comparing B73 vs. Mo17. Three biological replicates were tested at each stage with similar results, however, only one representative replicate is shown here. Figure 2 shows localization changes in the amino acids between different developmental stages. For the purposes of this figure, only the six most abundant amino acids in the maize root tissue are shown. For B73, the early stage of root development (2.5–3 cm in length) has greater amino acid signal in the cortex. As the root develops, amino acid signals are the highest abundance at 6–7 cm in length with growing abundance at the center of the root; then, at the length of 10–11 cm, there is a significant reduction of amino acid signals, especially in the cortex. A possible explanation for this is that in the early stages of development, amino acids are synthesized in the outer cortex of the root and as the plant develops, it begins to rely more on the transportation of amino acids and/or nitrogen from other parts of the plant or the surrounding environment. These ideas are supported by evidence in literature indicating that certain amino acids flow to the root from other parts of the plant (Oaks, 1966). Another interesting note is that the intermediate root (6–7 cm in length) has a higher abundance of amino acids overall than the earlier or later stages. While there is quite a bit of biological variation between roots at the same stage of development, the mid-length roots have higher signal intensities for the derivatized amino acids on average. This is especially apparent for the B73 roots. Supplementary Figure S2 in the supplementary information compares the average absolute signal intensities for the six most abundant amino acids at the different developmental stages.

In the case of Mo17 maize roots (right panel of Figure 2), the localizations are similar to that of B73 in the first two development stages. Both have the highest abundance of amino acid signal in the cortex of the root in the early stage (2.5–3 cm in length). In addition, they have a more or less even distribution between the center of the root and the cortex in the intermediate stage (6–7 cm in length). The major difference in the two genotypes occurs at the later stage of development (10–11 cm in length). While B73 loses amino acid signals in the cortex as they become concentrated to the pith and xylem, Mo17 does the opposite. Mo17 loses amino acid signals at the center of the root while those at the cortex remain similar. This suggests that B73 and Mo17 may have differences in the synthesis and/or transportation of amino acids during root development, especially at the later stage.

One hypothesis to explain the differences in localization at different developmental stages is that there is some gradient of amino acids along the length of the root, causing the localization to be different depending on the height. As a proof of concept, B73 maize roots at a later stage of development (16 cm in length) were embedded and sectioned at several different points along the length of the root. A representative data set is shown in Supplementary Figure S3. All amino acids are localized to the cortex near the root tips (11 or 15 cm distance from seed), similar to the early stage of root development (2 cm cross-section for 2.5–3 cm in length in Figure 2). However, they are more or less evenly distributed at the mid height (5 or 8 cm distance from seed), similar to an intermediate stage (2 cm cross-section at 6–7 cm in length in Figure 2). Finally, some amino acids, especially leucine/isoleucine and glutamine, are primarily localized to the center of the root at the 2 cm position near the seed, similar to the later stage of root development cross-sectioned at 2 cm from the seed for the root length of 10–11 cm in Figure 2. Namely, there are three distinguished stages of root development in terms of amino acid localization; the early stage near the tip of the root, the mid-stage at the middle of root, and the later stage near the seed. Genotypic differences between B73 and Mo17 are minimal for the first two stages as shown in Figure 2, but apparent in the last stage. The cross-section 2 cm from the seed at a later stage of development was therefore used in the further study to compare their differences with the hybrids.

### Genotypic Differences in Amino Acid Abundance and Localization

**FIGURE 3:**
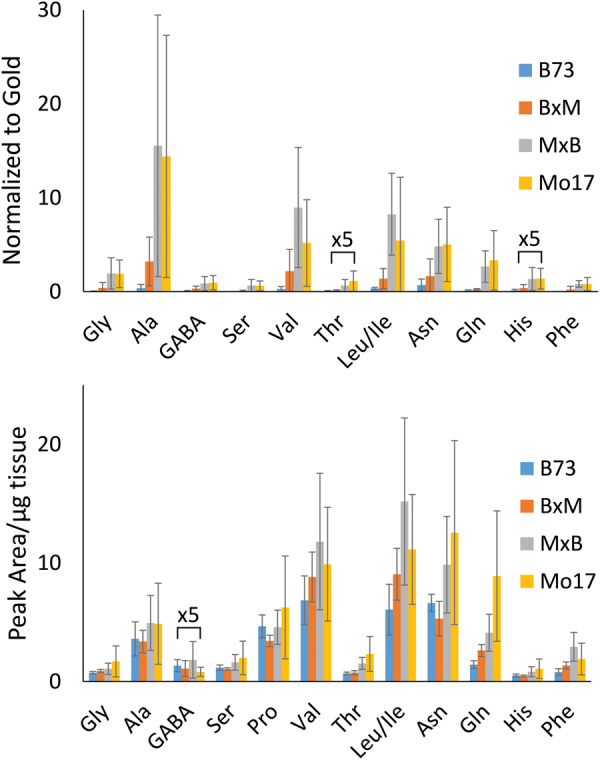
Comparison of amino acid abundance in each genotype analyzed by MALDI-MSI of tissue section compared to LC-MS extract. MALDI-MS data presents signal intensities for selected amino acids normalized to gold matrix peak (*n* = 3). LC-MS data presents integrated peak areas for the extracted ion chromatogram of each amino acid normalized to the tissue weight (*n* = 4).

The hybrid roots were compared to the parents at 10–11 cm in total length cryosectioned at 2 cm from the seed. Separate B73 and Mo17 plants were grown and processed at the same time to minimize experimental variation. Signal intensities for the most abundant amino acids are displayed in Figure 3 for each of the maize genotypes. These are the signal intensities averaged across the root areas in three biological replicates. There is a lot of biological variability in the abundance of amino acids even for the same genotype, making it difficult to determine how significant differences are; however, overall B73 and BxM have much lower amino acid signals compared to MxB and Mo17. Regardless of the genotype, alanine, valine, leucine/isoleucine, asparagine, and glutamine are the most abundant amino acids in maize root tissues. To confirm these findings with a more quantitative method, CA derivatized amino acids were also measured using LCMS for a similar region of the roots, harvested and extracted from 0 to 4 cm portion of the roots at 10–11 cm in total length. The genotypic differences are much less apparent in LCMS data due to the concentration gradient of amino acids along the root length (as seen in Supplementary Figure S3). The general trend of LCMS results, nonetheless, corroborated the MALDI-MSI findings; the same amino acids were found to be the most abundant and Mo17 and MxB have higher average signal levels than B73 and BxM. The LCMS data did include derivatized proline, which was detected with good signal intensity, unlike the MALDI-MSI data. We hypothesize that the on-tissue reaction might be too slow for secondary amines and does not provide high enough yield with the limited reaction time, unlike the in-solution reaction used for LCMS.

**FIGURE 4:**
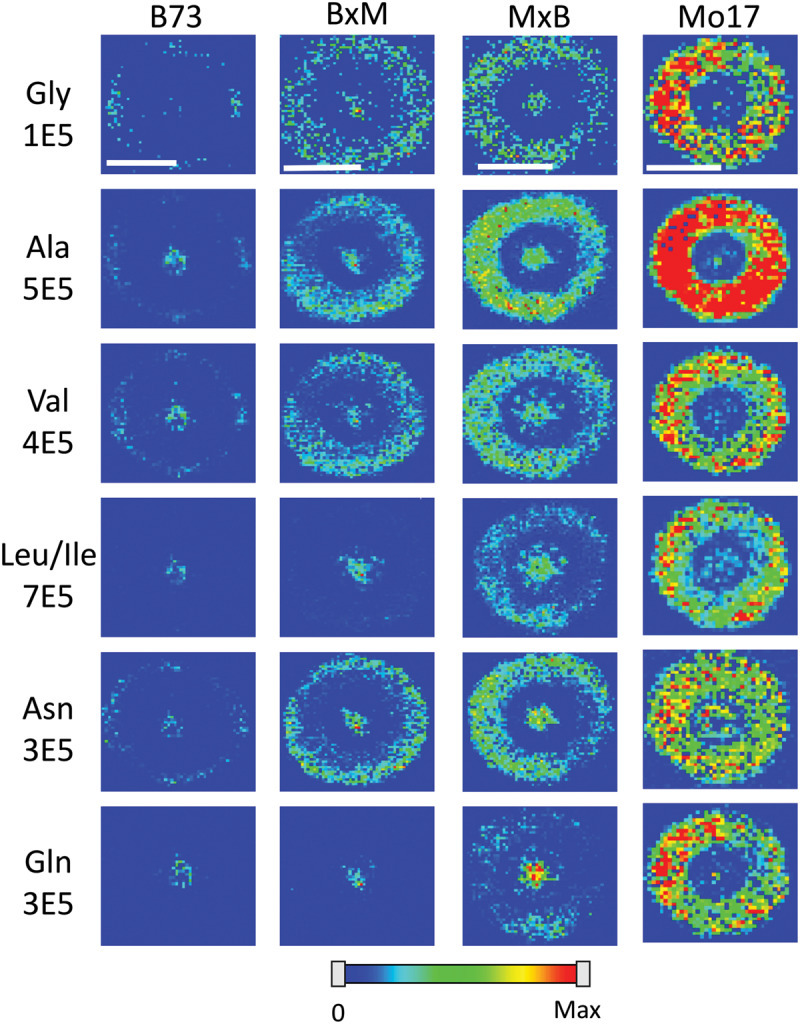
MS images of the six most abundant amino acids in B73, BxM, MxB, and Mo17 maize roots cryosectioned at 2 cm below the seed for the development stage of 10–11 cm length. The numbers below each amino acid label correspond to the maximum intensity scale used to produce false color image. The scale bar is 500 μm.

Figure 4 shows representative MS images of the six most abundant amino acids detected in maize roots (10–11 cm in length or about 8–10 days old, embedded and cryosectioned about 2 cm away from the seed) across all four genotypes. The same intensity scale is used for MS images of each amino acid to allow for a fair comparison of the relative abundances between the genotypes. Similar to the signal intensities in Figure 3, the MS images show that Mo17 has much higher amino acid signal than B73 and the hybrids each have signal levels similar to that of the maternal parent. In addition to the obvious differences in overall signal and abundance, there are also more subtle differences in localization of amino acids between the two inbred lines, similar to Figure 2. In Mo17, most amino acids have the highest signal intensity in the cortex and minimal signal in the pith and xylem, with asparagine as a possible exception. In contrast, B73 has much of the amino acid signal localized to the center of the root and very little in the cortex, except for glycine and asparagine, which have a more even distribution. In fact, leucine/isoleucine and glutamine have almost no signal intensity in the cortex of B73. Interestingly, similar localizations are found in the hybrids, which mostly follow maternal inheritance; MxB obtains characteristics relating to amino acid localization from Mo17, whereas BxM obtains characteristics from B73. For example, leucine/isoleucine and glutamine are mostly localized at the center for BxM while significantly present in the cortex for MxB. Another example of possible maternal inheritance is the increased amino acid signal in MxB over BxM. This evidence for maternal inheritance is similar to the previous finding that the localization of thylakoid membrane lipids, specifically phosphatidylglycerols (PGs), in maize hybrids also follow characteristics of the maternal parent (Dueñas et al., 2019). Despite this example of maternal inheritance, there are other factors suggesting the hybrids inherit some characteristics from the paternal parent as well. For example, the increased abundance of amino acids in the center of the roots in MxB compared to Mo17 suggests characteristics of B73 in MxB.

**FIGURE 5:**
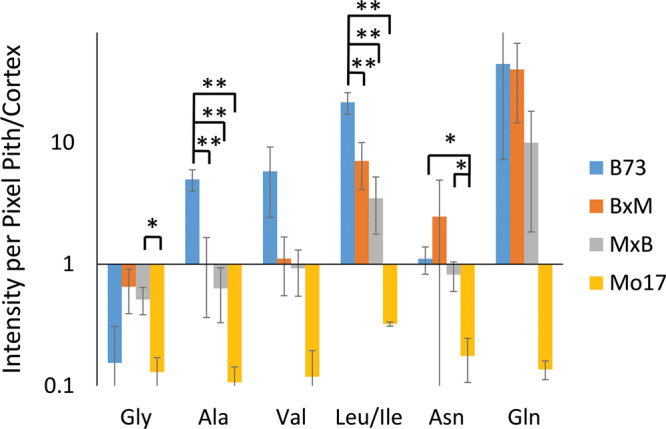
The ratio of amino acid signals in the pith vs. cortex per unit area (*n* = 3). The ^∗^ and ^∗∗^ indicate a *p* < 0.05 and < 0.01, respectively. The y-axis is on a logarithmic scale with the x-axis crosses the value of one, where the pith and cortex have the same signal intensity per unit area.

Some may argue that these differences in localization are simply due to changes in ionization efficiency for different portions of the root if chemical composition is somewhat different between the genotypes. To address this issue, one root of each genotype was sprayed with an internal standard, deuterated alanine, and the images were created for several amino acids normalized to the deuterated alanine peak. As shown in Supplementary Figure S4, localizations look the same after this normalization suggesting there is no significant difference in ionization efficiency between different parts of the tissue. The localization differences described above are more apparent in Figure 5, which shows the signal intensity for amino acids in the pith normalized to the signal intensity in the cortex for each root. These intensities are also normalized to the overall size of the pith or cortex to account for the different tissue areas; namely, (Σ_Pith_I/Area_Pith_)/(Σ_Cortex_I/Area_Cortex_). The y-axis of this figure is in a logarithmic scale and a ratio of one, located where the x-axis crosses the y-axis, indicates an even distribution of amino acids. With the exception of glycine, which is more abundant in the cortex than the pith in every genotype, B73 has ratios above one for every amino acid and Mo17 has ratios below one. For the most part, the hybrids have ratios in between those of the two parents, however, to varying degrees. For example, both hybrids have ratios close to one for alanine, valine, and asparagine, indicating a fairly even distribution. However, for leucine/isoleucine and glutamine, the hybrids both have ratios greater than one, but lower than that of B73. Some high standard deviations associated with asparagine and glutamine are likely due to the dramatic changes in localization along the length of the root for the two amino acids as shown in Supplementary Figure S3. Amino acids that have less biological variability exhibited some statistically significant differences. For example, the differences observed between B73 and each of the other genotypes are statistically significant for alanine and leucine/isoleucine with *p* < 0.01. For asparagine, the difference is significant only when comparing B73 to Mo17 or MxB to Mo17 with a *p* < 0.05. Overall, Figure 5 nicely displays how the localization of most amino acids in the hybrids is a blended inheritance from both parents. The intermediate normalized intensities observed for alanine, valine, leucine/isoleucine, and asparagine especially correspond to more evenly distributed amino acids in the pith and cortex compared to the parents.

Amino acids localized primarily in the center of the root, in the pith and xylem, such as leucine/isoleucine and glutamine in B73 and BxM, may suggest these amino acids are transported to the root from other parts of the plant as opposed to being synthesized in the root itself. This would be consistent with the pith and xylem being the cells primarily responsible for the transportation of nutrients (Tegeder and Hammes, 2018). Mo17 has very little amino acid signal in the pith and xylem compared to the other maize genotypes. Therefore Mo17 may be synthesizing more amino acids in the root cortex itself and rely less on the transport of amino acids from other parts of the plant or the environment. Previous research has shown that many of the enzymes necessary for synthesizing amino acids are located in plastids and plastids achieve their highest level of development in cortical tissue (Miflin, 1974; Whatley, 1983). This supports the hypothesis that amino acids are being synthesized in the root cortex.

### Changes in Amino Acid Abundance and Localization in Nitrogen Rich Conditions

**FIGURE 6:**
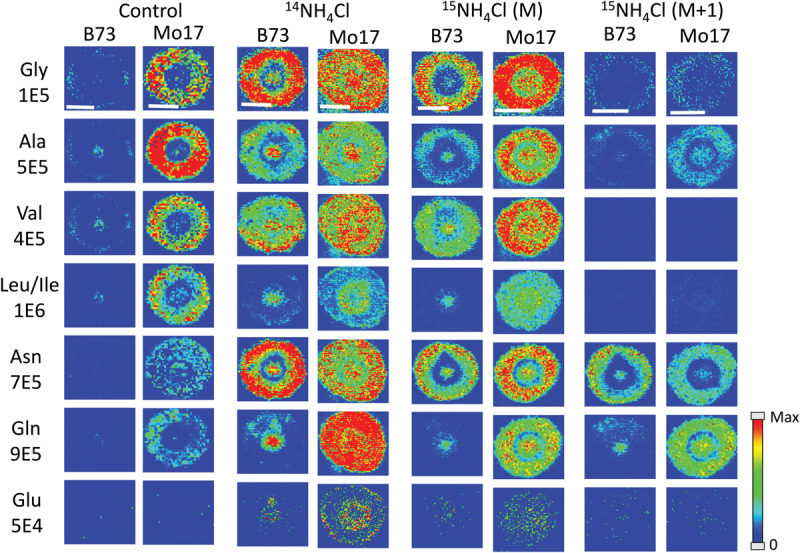
MS images of selected amino acids in B73 and Mo17 maize root sections grown in 10 mM ammonium ion, either ^14^NH_4_Cl or ^15^NH_4_Cl, compared to the control grown in water. The first three sets of images are of the monoisotopes (M peak) containing only ^12^C and ^14^N. The last set is of the isotope (M + 1 peak) from ^15^NH_4_Cl data containing one isotope of either ^15^N or ^13^C. The root sections were made at 2 cm below the seed when the root reached the length of 10–11 cm. The numbers below each amino acid label correspond to the maximum intensity scale used to produce false color image. The scale bar is 500 μm.

All of the data shown up to this point has been from maize roots grown in pure water, which is nitrogen deficient compared to growing in soil. As plants are typically grown using fertilizer for a source of nitrogen, roots were also grown in a 10 mM solution of ammonium chloride, which acts as a nitrogen source during root development. Figure 6 displays the representative MS images of B73 and Mo17 maize roots from three replicates grown in ammonium chloride compared to water. Not surprisingly, overall signals are higher for both B73 and Mo17 with the addition of ^14^NH_4_Cl. Mo17 roots show changes in the localization when grown under nitrogen rich conditions with a more even distribution across the anatomy of the root. Rich nitrogen absorbed from outside might encourage the transportation of amino acids out of the roots once they are synthesized in the root cortex. In addition, the signal difference for amino acids between B73 and Mo17 maize is not as apparent when grown in the nitrogen containing solution compared to water, with the possible exception of glutamine. We hypothesize that B73 has a higher abundance of amino acids in the center of the root compared to the cortex because it relies on transportation from other parts of the plant; therefore, additional nitrogen provided from the environment may allow the B73 maize to synthesize more amino acids than would otherwise be possible. It is known that plants grown in nitrogen deficient conditions have less abundant amino acids in the roots and an altered proteome that will impact amino acid metabolism (Møller et al., 2011). Interestingly, leucine/isoleucine and glutamine are still highly enriched at the center of the B73 roots, potentially suggesting that pathways involving these amino acids are not affected by the availability of external nitrogen. Another interesting note is the increased abundance of glutamate by two orders of magnitude, which is almost invisible without additional nitrogen. This is consistent with glutamate being important for nitrogen assimilation in roots (Fentem et al., 1983).

As there are two different nitrogen sources (transported from other parts of the plant, and absorbed from environment), we performed an experiment to determine whether we can distinguish the two by growing roots in 10 mM ^15^N-ammonium chloride. This will determine which amino acids take up nitrogen from the environment most efficiently during development. Images from this experiment are also available in Figure 6 (two sets of images to the right). One would expect the images of amino acids with ^14^N (labeled M in the figure) to be similar to the control because the nitrogen from the environment is ^15^N and would appear as an isotope peak at M + 1. However, these root images look much more like the ones grown in ^14^N ammonium chloride than the ones grown in water. This suggests that the plant does not process environmental nitrogen any different from nitrogen already present in the seed. Instead, additional nitrogen (or ammonium ion) from the environment may trigger some biological pathway that does not operate under nitrogen deficient conditions. The mass resolution of the instrument used was not high enough to resolve peaks with ^13^C- vs. ^15^N-amino acids. As shown in Supplementary Figure S5, the isotope of derivatized glutamine appears as a single peak for ^13^C_1_- or ^15^N_1_-isotope at “M + 1” position and ^13^C_2_-, ^15^N_2_-, or ^13^C_1_^15^N_1_-isotope at “M + 2” position. As the ^13^C and ^15^N peaks were not resolved, the M + 1 peak contains both ^13^C_1_- and ^15^N_1_-gluatamine peaks. Considering the natural abundance of ^13^C and ^15^N, M + 1 peak in the maize root grown in ^15^N-ammonium solution shown in Supplementary Figure S5 is made up of ∼90% ^15^N from external ^15^N-ammonium ion. The final two columns in Figure 6 show the amino acids synthesized from nitrogen in the environment, thus ^15^N-labeled and composing most of the M + 1 peak. They show similar localization with the ^14^N-labeled M peak, amino acids synthesized from nitrogen already present in the seed. This provides further support for our hypothesis that environmental nitrogen only triggers a mechanism that affects abundance and localization of amino acids but does not differentiate between internal vs. external nitrogen. Some of the detected amino acids significantly incorporated ^15^N, especially asparagine and glutamine, which is not surprising considering they are known to play the key role in nitrogen assimilation (Fentem et al., 1983).

**FIGURE 7:**
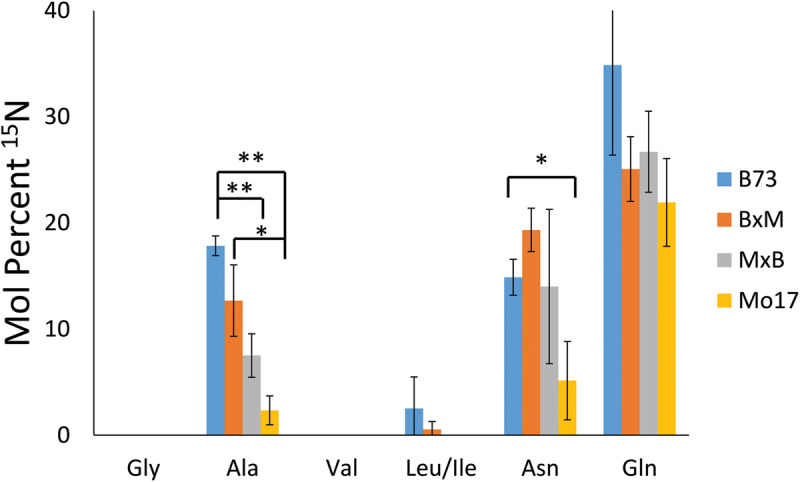
Mol percent of ^15^N incorporation into amino acids of maize roots grown at 10 mM ^15^N-ammonium ion and cryosectioned at 2 cm below the seed at the root length of 10–11 cm. The error bars come from three biological replicates. The * and ** indicate a *p* < 0.05 and < 0.01, respectively.

To explore how much ^15^N-labeled external nitrogen is incorporated for each amino acid, the peaks corresponding to each of the labeled amino acids were deconvoluted using an Excel spreadsheet developed by Gruber et al. (2007) considering the natural abundance of the isotope. The relative level of the incorporated ^15^N from three biological replicates of each genotype are summarized in Figure 7. These reported results represent the percent of nitrogen in each amino acid that has come from environmental ^15^N. Glutamine has the highest level of ^15^N incorporation (22–35%) followed by asparagine and alanine (5–19% and 2–18%, respectively). Glutamine and asparagine are well known for nitrogen storage (Hildebrandt et al., 2015), so they would take up nitrogen from the surrounding environment and store it throughout the early development of the plant. Alanine also has been known to shuttle nitrogen between cells in crop plants, which could explain its environmental nitrogen uptake (Shrawat et al., 2008). In contrast, other amino acids, such as glycine, valine, and leucine/isoleucine, did not incorporate any appreciable amount of nitrogen from the environment. In a much later stage of development, a majority of nitrogen would be eventually replaced by ^15^N in every amino acid; however, at this stage of development, most nitrogen still seems to be coming from the seeds except in the case of the three amino acids related with nitrogen storage or transportation.

In addition to the differences between amino acids, there also are some differences depending on the genotype of maize. There is significantly less incorporation of ^15^N in the three amino acids in Mo17 maize than in B73, with the possible exception of glutamine. *P*-values associated with the environmental nitrogen uptake for alanine and asparagine are <0.01 and <0.05 respectively when comparing B73 and Mo17. As noted earlier, Mo17 has much higher amino acid content in seedlings (Fentem et al., 1983) and the localization of amino acids in Mo17 roots indicates it could be synthesizing most of the necessary amino acids directly in the cortex of the root. This may be the reason Mo17 is not taking in as much nitrogen from the nutrient solution in the surrounding environment. For the most part, the nitrogen incorporation in the hybrids was in between that of the two parents, although statistically insignificant due to the large error bars, with the exception of alanine. This is further evidence that the hybrid maize has blended traits from the two inbred lines and benefits from characteristics of both parents.

## Conclusion

In this study, MALDI-MSI combined with CA chemical derivatization was utilized to study amino acids in maize root tissue. New insight into the localization of amino acids throughout the growth and development of maize seedlings as well as inheritance patterns of hybrid maize was observed that would not have been possible without this method. The surface imaging allowed for previously unknown differences in relative quantification between different parts of the maize root with relatively little sample preparation compared to LCMS and gas chromatography (GC)-MS. In contrast to MALDI-MSI, LC, and GCMS both require lengthy extractions, resulting in the destruction of the sample and inability to obtain spatial information. Although this study highlighted the use of MALDI-MSI for the analysis of free amino acids, the technique is versatile so other classes of compounds can be detected and visualized in the same sample. This approach, however, comes with its own limitations, most notably the difficulty in quantification compared to other techniques. An additional disadvantage due to the derivatization is the possibility of side reactions, i.e., CA reacting with compounds other than primary amines. The latter makes utilizing this technique for untargeted analyses challenging, but it may be overcome through systematic study in side reactions and more selective reagents.

While the overall abundance and localization changed in different developmental stages as well as at different points along the length of the root, locations near the seed (∼2 cm away) displayed major differences between genotypes at later stages of development. Specifically, B73 had lower amino acid abundance and showed a localization primarily in the center of the root for most amino acids. In contrast, Mo17 had higher abundance and amino acids were mostly localized to the root cortex. Both the hybrid roots grown and prepared in the same way as the parents had abundance levels that were similar to that of the maternal parent. BxM had abundance similar to or slightly higher than B73, but much lower than MxB or Mo17. Likewise, MxB had signal levels similar to Mo17. In terms of localization, a blended inheritance was observed for most amino acids, as noted by the pith/cortex signal intensity ratios, with the exception of glycine. Alanine and valine provided the best examples of blended inheritance with B73 having a signal ratio much greater than one, indicating localization primarily to the pith, Mo17 having a ratio much less than one, indicating localization primarily to the cortex, and both hybrids having a ratio close to one, indicating an even distribution. Comparing the characteristics relating amino acids in inbred lines of maize to their reciprocal hybrids gave insight into hybrid inheritance patterns. In addition, understanding the changes in amino acid localization and abundance provides essential information relating to the transportation of nutrients in the early stages of plant development.

Differences in amino acid localization and abundance were also observed for roots grown in nitrogen rich conditions compared to those grown in water. The addition of a nitrogen source in the form of an ammonium ion to the growth environment caused changes such as lessened signal discrepancy for amino acids between B73 and Mo17 compared to nitrogen deficient conditions, and more uniform distribution of amino acids, especially in Mo17. An isotope labeling experiment using ^15^NH_4_Cl provided some insight about nitrogen uptake from environment. First of all, there is no difference between the localization of ^14^N-amino acid and ^15^N-amino acid, regardless of whether they are grown in ^14^NH_4_Cl or ^15^NH_4_Cl. This suggests the change in the abundance and localization due to the ammonium ion is the result of triggering a new mechanism or biochemical pathway, and not due to different distributions of nitrogen from environment. When comparing the relative amount of ^15^N uptake for each amino acid, only glutamine, asparagine, and alanine incorporated a significant amount of nitrogen from the environment in this early stage of root development, consistent with their major roles in nitrogen storage and transport. In terms of the amount of ^15^N incorporation, B73 and Mo17 show the most and the least incorporation, respectively, the two hybrid maize have an intermediate level of ^15^N incorporation between the two parents, which provides further evidence that the characteristics of the hybrids are inherited from both parents. These experiments can help shed light on nitrogen assimilation and nitrogen use efficiency in maize. Studying amino acids in maize roots has not only given insight into differences between maize genotypes and inheritance patterns, but has also shown how amino acids accumulate throughout roots as they grow and develop. Considering the success of this method for the current application, there is room to expand this work for additional applications in the future. For example, amino acids such as phenylalanine and tyrosine could be related back to the localization of defense compounds derived from the phenylpropanoid pathway.

## Data Availability Statement

The raw data supporting the conclusions of this article will be made available to any qualified researcher without any reservations.

## Author Contributions

KO and YL designed the experiment. KO did all of the sample preparation and data collection for the experiment. Data was analyzed by KO with the help and guidance of YL. The manuscript was prepared and written by KO and YL.

## Conflict of Interest

The authors declare that the research was conducted in the absence of any commercial or financial relationships that could be construed as a potential conflict of interest.
