# Supplementary material for: Visualizing Genotypic and Developmental Differences of Free Amino Acids in Maize Roots With Mass Spectrometry Imaging
Source: Front Plant Sci. 2020 May 25;11:639. doi: 10.3389/fpls.2020.00639 (PMC7261921; doi:10.3389/fpls.2020.00639)
Supplement: Supplementary file 1 [file Data_Sheet_1.pdf]

Supplementary Material

1 Supplementary Figures

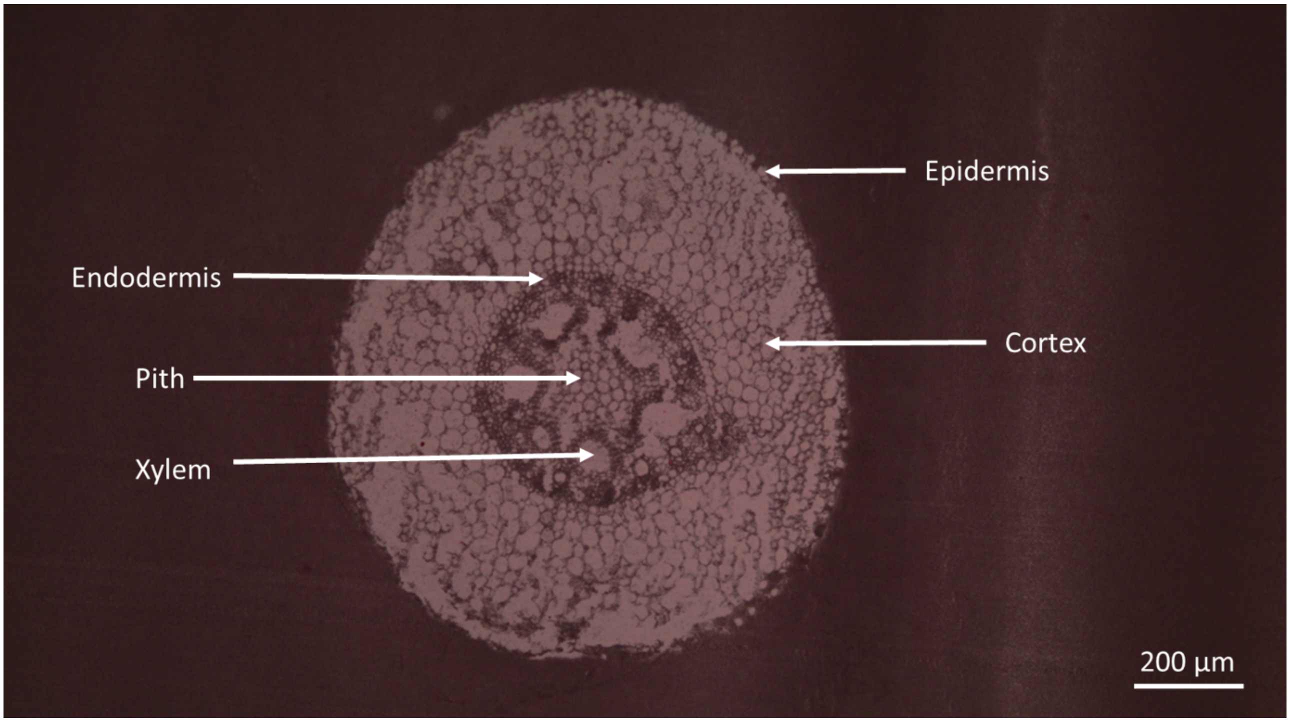

Figure S1. Anatomy of a B73 maize root.

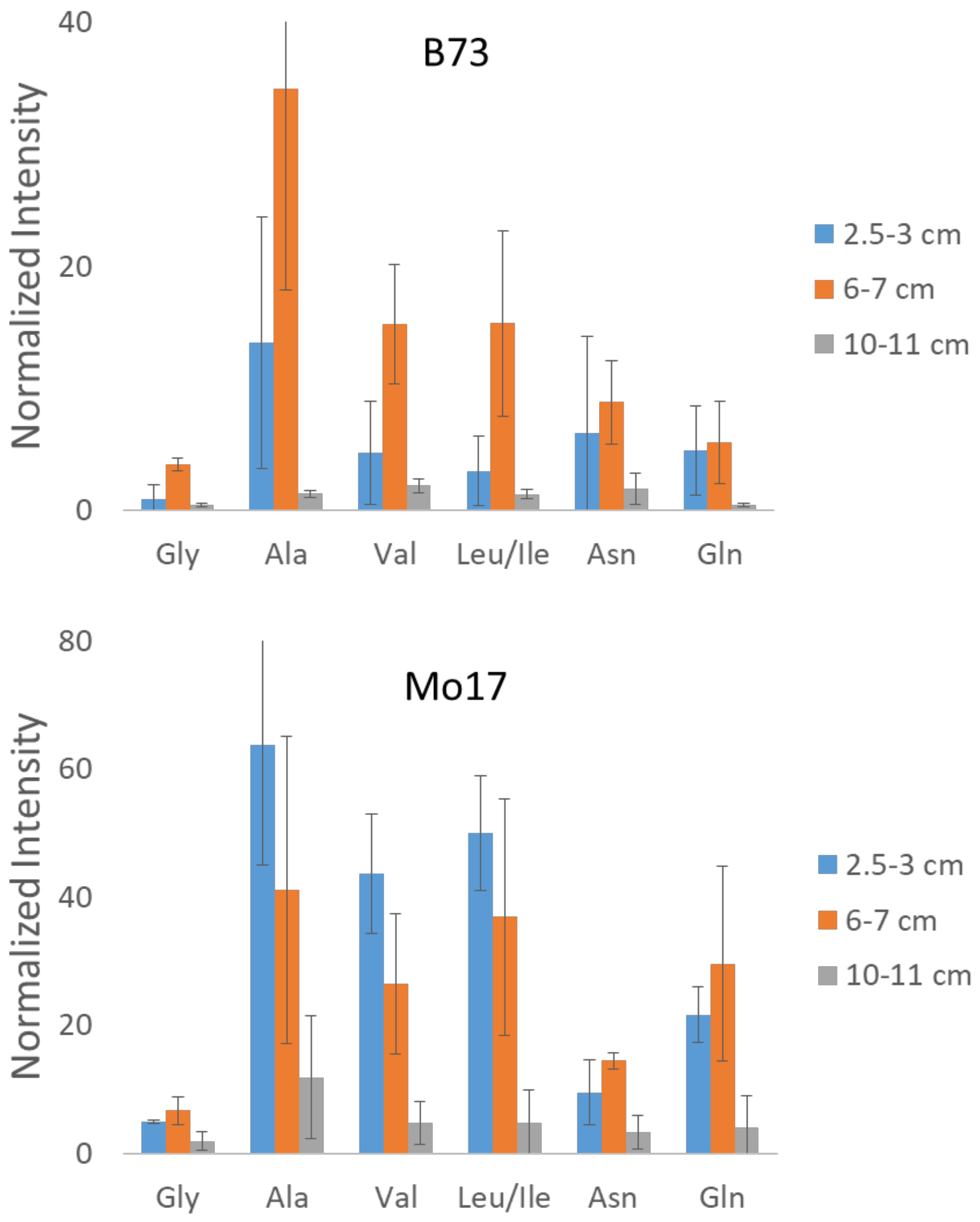

Figure S2. Signal intensities for the six most abundant amino acids in B73 and Mo17 maize at different developmental stages normalized to gold matrix peak. Normalized intensities come from MS images in Figure 2 (cryosectioned 2 cm away from the seed regardless of the stage of development). Error bars are the standard deviations from three replicates.

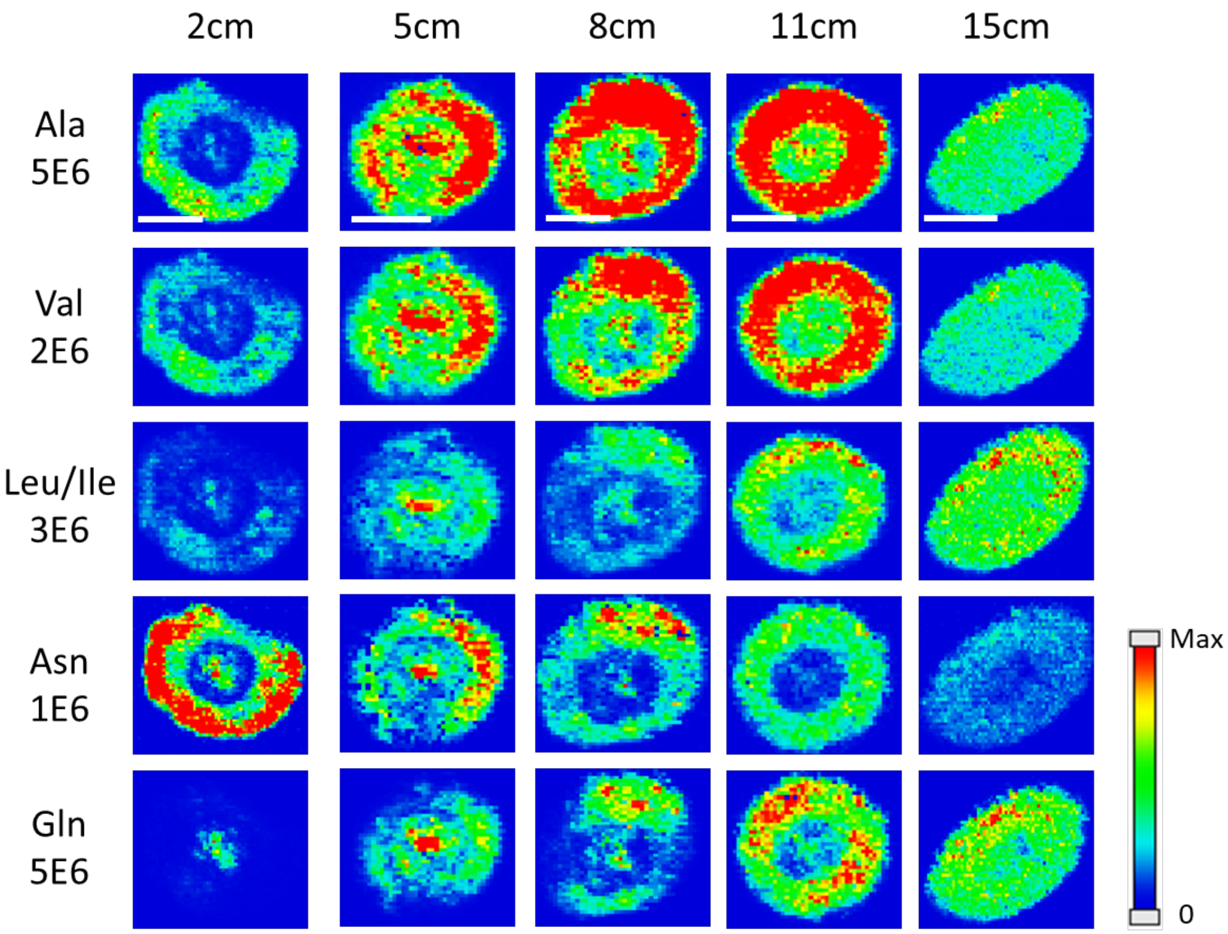

Figure S3. MS images of selected amino acids for B73 maize root at 16 cm in length, grown in water, embedded and sectioned at several different positions along the length of the root. The numbers below each amino acid label correspond to the maximum intensity scale used to produce false color image. The scale bar is 500 µm.

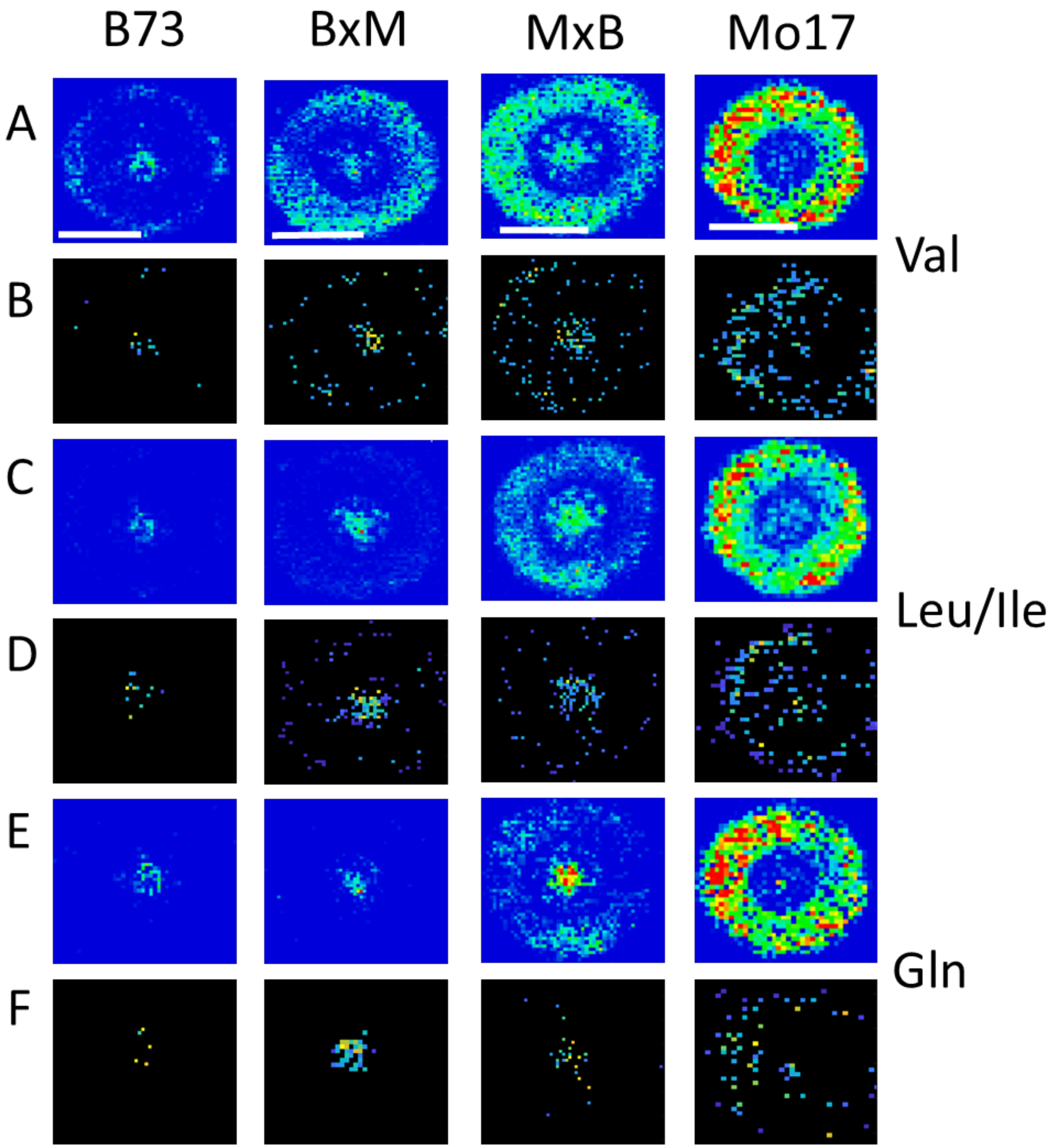

Figure S4. Localization of the amino acids, valine (**A, B**), leucine/isoleucine (**C, D**), and glutamine (**E, F**), with and without normalization to deuterated alanine internal standard. Images in rows labelled A, C, and E are neither sprayed with d-Ala nor normalized, while those in rows B, D, and F are normalized to d-Ala signals after sprayed with d-Ala. The scale bar is 500 µm.

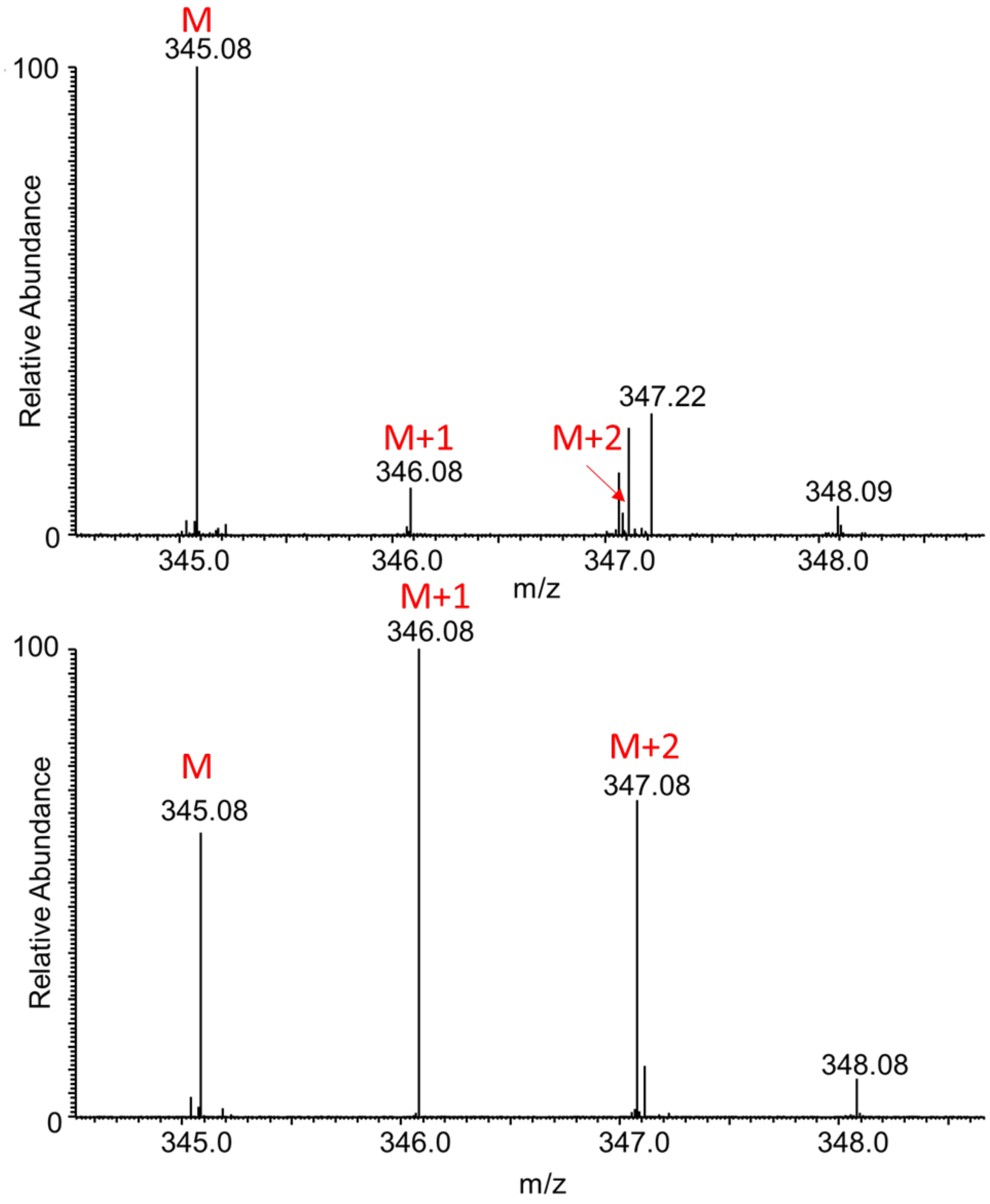

Figure S5. Mass spectra for B73 maize roots grown in water (top) and 10 mM ^{15}N ammonium chloride (bottom) zoomed into the peaks representing derivatized glutamine.
